# Linking GABA and glutamate levels to cognitive skill acquisition during development

**DOI:** 10.1002/hbm.22921

**Published:** 2015-09-09

**Authors:** Kathrin Cohen Kadosh, Beatrix Krause, Andrew J. King, Jamie Near, Roi Cohen Kadosh

**Affiliations:** ^1^ Department of Experimental Psychology University of Oxford Oxford United Kingdom; ^2^ Department of Psychology Institute of Psychiatry King's College London London United Kingdom; ^3^ Department of Physiology, Anatomy and Genetics University of Oxford Oxford United Kingdom; ^4^ Department of Psychiatry McGill University Montreal Canada

**Keywords:** magnetic resonance spectroscopy, cognitive development, γ‐aminobutyric acid, glutamate, cortical excitability, face processing, working memory

## Abstract

Developmental adjustments in the balance of excitation and inhibition are thought to constrain the plasticity of sensory areas of the cortex. It is unknown however, how changes in excitatory or inhibitory neurochemical expression (glutamate, γ‐aminobutyric acid (GABA)) contribute to skill acquisition during development. Here we used single‐voxel proton magnetic resonance spectroscopy (^1^H‐MRS) to reveal how differences in cortical glutamate vs. GABA ratios relate to face proficiency and working memory abilities in children and adults. We show that higher glutamate levels in the inferior frontal gyrus correlated positively with face processing proficiency in the children, but not the adults, an effect which was independent of age‐dependent differences in underlying cortical gray matter. Moreover, we found that glutamate/GABA levels and gray matter volume are dissociated at the different maturational stages. These findings suggest that increased excitation during development is linked to neuroplasticity and the acquisition of new cognitive skills. They also offer a new, neurochemical approach to investigating the relationship between cognitive performance and brain development across the lifespan. *Hum Brain Mapp 36:4334–4345, 2015*. © **2015 The Authors. Human Brain Mapping Published byWiley Periodicals, Inc.**

**Abbreviations**
BOLD: blood‐oxygenated level dependent
CFMT: Cambridge memory test for faces
CSF: cerebrospinal fluid
FG: fusiform gyrus
FMRIB: functional MRI of the brain
GABA: γ‐aminobutyric acid
^1^H‐MRS: proton magnetic resonance spectroscopy
IPS: intraparietal sulcus
MPRAGE: magnetization prepared rapid gradient echo
NAA: *N*‐acetyl aspartate
SNR: signal to noise ratio
VOI: voxels of interest
VS‐WMT: Visuospatial working memory task

## INTRODUCTION

Our current approach to understanding human development is based mainly on observations of how changes in cognitive performance relate to functional and anatomical brain development [Cohen Kadosh, et al., 2013a; Shaw, et al., 2006]. Despite the importance of changes in excitatory and inhibitory inputs for the maturation of cortical circuits [Carcea and Froemke, 2013; Hensch and Bilimoria, 2012; Hensch and Stryker, 2004], nothing is known about the relative contribution of neurotransmitter interactions, such as excitatory (glutamate) or inhibitory (γ‐aminobutyric acid, (GABA)) neurotransmitter levels, to these developmental changes in humans. Here we used single voxel proton magnetic resonance spectroscopy (^1^H‐MRS) to reveal how differences in neurotransmitter concentrations in several brain regions relate to cognitive proficiency in face processing and visuospatial working memory skills in children and adults.

Neural face processing is one of the best‐studied tasks performed by the human brain. This makes it an ideal model for investigating the mechanisms of cortical specialization of cognitive functions, especially as it has been shown that children will require 10 years or more of practise to identify faces with adult‐like proficiency [Cohen Kadosh, 2011a; Durand, et al., 2007; Karayanidis, et al., 2009; Mondloch, et al., 2002, 2003]. In the mature brain, face processing relies on a core network of brain regions, including the inferior occipital gyrus (IOG), the fusiform gyrus, and the superior temporal sulcus [Cohen Kadosh, et al., 2010; Haxby, et al., 2000; Ishai, 2008]. Recent progress in developmental neuroimaging has provided valuable insights into the emerging face networks in the brain [Cohen Kadosh, 2011b; Cohen Kadosh and Johnson, 2007]. For example, it has been shown that children show face‐preferential activation in core network regions from an early age [Cantlon, et al., 2011; Cohen Kadosh and Johnson, 2007; Cohen Kadosh, et al., 2013a]. However, activation in additional brain regions, such as the left inferior frontal gyrus (IFG) in children, but not in adults, has also been reported in several independent studies [Gathers, et al., 2004; Passarotti, et al., 2007; Scherf, et al., 2007].

There is some evidence for a relationship between cognition and GABA or glutamate measured by MRS, but it is not known how these relationships emerge during development [Jocham, et al., 2012; Majdi, et al., 2009; Sandberg, et al., 2014; Sumner, et al., 2010; Terhune, et al., 2014, in press]. In particular, it is currently unclear how changes in the balance between excitatory and inhibitory neurotransmitters (E/I balance) contribute to the maturation of functional networks [Cousijn, et al., 2014; Muthukumaraswamy, et al., 2012].

E/I balance is a fundamental property of adult cortical microcircuitry, but is likely to be dynamic during development [Atwood and Wojtowicz, 1999; Lehmann, et al., 2012], possibly as a result of changes in the way glutamatergic and GABAergic inputs interact with the intrinsic membrane properties of neurons [Ben‐Ari, et al., 2007]. While maintaining an appropriate balance between excitation and inhibition appears to be crucial for normal brain functioning, developmental changes in both glutamatergic and GABAergic mechanisms are thought to underlie the experience‐dependent plasticity of cortical circuits and are deficient in a variety of neurodevelopmental disorders [Murphy, et al., 2005; Ramamoorthi and Lin, 2011]. This raises the possibility that the developmental changes in cortical neurochemical balance affect learning and the acquisition of new cognitive skills, such as face processing.

To combine these currently distinct research tracks in the fields of cognition, brain development, and neurotransmitter expression, we investigated in this study the relationship between cortical glutamate/GABA ratios and cognitive processing proficiency in three cortical regions in a group of young children and a group of young adults. Specifically, we compared glutamate/GABA ratios and cognitive performance changes as a function of development in the IFG, an early face network region [Johnson, et al., 2009], the IOG, a core region from the mature face network [Haxby, et al., 2000], and the intraparietal sulcus (IPS), a region not usually involved in face processing tasks. The latter region served both as a control region for the face processing task, but also as a target region for a visuospatial working memory assessment, an approach which allowed us to investigate the domain‐specificity of the observed effects. This dual task approach was based on the assumption that studying face processing alone will not allow us to draw strong conclusions as to whether any observed relationships between cognitive development and neurochemical expression are specifically related to this function. Visuospatial working memory abilities represent an excellent control condition, as previous research has highlighted that these improve continuously during childhood and adolescence, while relying on a distinct network of parietal, occipital, and frontal regions, which differs from the core and extended face processing network [Haxby, et al., 2000; Klingberg, 2006; Stokes, et al., 2009].

We note that whereas previous studies have shown an association between the BOLD (blood‐oxygenated level dependent) response in fMRI and neurotransmitter levels (e.g., GABA or glutamate) [Hu, et al., 2013; Muthukumaraswamy, et al., 2009, 2012; Stagg, et al., 2014] other did not [Cousijn, et al., 2014; Harris, et al., 2015], and the exact source of GABA and glutamate, as measured by MRS, therefore appears to be complex and currently unresolved [Stagg, et al., 2011]. Specifically, the relative contributions from intracellular (metabolic) or extracellular (neutrotransmitter) pools to the obtained MRS spectrum from a specific brain region remain to be determined. For the purpose of the current study, we will therefore, in a first step focus on how differences in the ratio of glutamate to GABA relate to cognitive functioning, with the aim of providing an additional measure to understanding developmental changes in brain functioning.

## MATERIALS AND METHODS

### Participants

Twenty‐eight subjects, 14 adults (average age: 21.6 years, SD: 1.03 years, range 20–23 years) and 14 children (average age: 8.9 years, SD: 0.74 years, range 7–10 years) participated in this study. An additional four children and one adult were recruited, but we excluded five participants because of severe scanner anxiety (two children), excessive motion during the scanning session (one child), and technical failure (one child, one adult). Finally, three adults showed strong extracranial lipid contamination in the IOG (because of the voxel having been placed too close to the scalp, and were therefore excluded from the correlational analyses for that region). All participants were right‐handed and female (to eliminate any variance due to gender at this stage), had normal or corrected to normal vision, and reported no history of neurological or mental illness. They received compensation (adults: £25, children: £20 national book token) for their participation in the experiment. The study was approved by the local ethics committee (University of Oxford) and each participant gave written informed consent (adults, and the child's primary caregiver) or informed assent (children) prior to testing.

### Behavioral Tasks

#### The Cambridge memory test for faces

We used the ***Cambridge memory test for faces (CFMT)*** [Duchaine and Nakayama, 2006] to assess face processing proficiency in both age groups. The CFMT is a computer‐based face recognition task, where participants are introduced to six target faces, which they then are required to recognize in a display of three faces (one of which is the target). For each target face, three test items (1) contain views identical to those studied in the introductory phase (CFMT‐1), (2) present novel views (CFMT‐2), and (3) present novel views with noise (CFMT‐3). Accuracy rates for each subscale (1–3) are then combined into a total accuracy rate (CFMT‐total) for each participant and the raw scores transformed into *z*‐scores. Test administration took no more than 15 minutes in all participants.

##### Visuospatial working memory task

We used the Corsi Block tapping task [Kessels, et al., 2000] to assess the visuospatial working memory span in our children and adult participants. The task consists of nine wooden cubes that are mounted on a board. The participant first observes the examiner tapping a sequence of blocks, which the participant then has to repeat in the correct sequence. Visuospatial working memory task ***(***VS‐WMT) can then be measured by increasing the length of the sequences. Testing stopped as soon as the participant failed on two subsequent sequences of the same length. Raw scores were derived from the total number of correctly repeated sequences and converted into *z*‐scores. Test administration took no more than 8 minutes for each participant.

#### Procedure

Each participant first underwent 1 hour of MRI scanning (three magnetic resonance spectroscopy sessions for the three brain regions (IFG, IPS and IOG) and 1 structural scan). All participants were then invited to participate in a separate behavioral testing session.

##### ^1^H‐MRS

**Figure 1:**
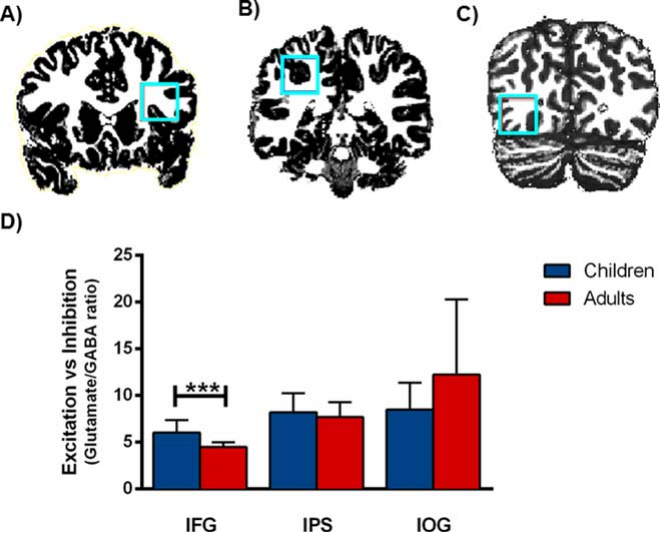
Cortical glutamate/GABA ratios in the child and adult brain per region. ^1^H‐MRS voxel (2x2x2cm) positions for (**A**) inferior frontal gyrus (IFG), (**B**) intraparietal sulcus (IPS), and (**C**) inferior occipital gyrus (IOG) are shown on coronal slices. **D**: Total quantities of the normalized glutamate/GABA ratios in the IFG, IPS, and IOG. Children showed significantly higher relative excitation levels than adults in the IFG. *** *P* <0.001. [Color figure can be viewed in the online issue, which is available at http://wileyonlinelibrary.com.]

We used a Siemens 3T Verio with a 32‐channel head coil to acquire magnetic resonance imaging data at the Oxford Centre for Functional MRI of the Brain (FMRIB). T1‐weighted MR images with 1 mm thick slices were acquired (MPRAGE; magnetization prepared rapid gradient echo) using TR = 2,040 ms, TE = 4.68ms, TI = 900ms (inversion time), and a flip angle of 8° for the voxel‐based morphometry (VBM) analyses and the planning of the MRS acquisition. After the acquisition, coronal, axial, and sagittal slices of 3 mm thickness were reconstructed and 2 × 2 × 2 cm^3^ voxels of interest (VOI) were then manually centered on axial and coronal slices over the right IPS, the left IFG, and the right IOG (Fig. 1). The IFG was placed based on coronal and axial images to cover the inferior frontal tissue without capturing temporal cortices. None of the voxels exceeded the located gyrus toward the anterior or posterior end. For the IPS, the hand knob of the motor cortex was identified on axial slices [Yousry, et al., 1997]. We centered the voxel on the sulcus posterior and inferior to this landmark for the motor cortex. The IOG was localized by angling the voxel along the outer cortical edge away from the calcarine fissure and as ventral as the size of the voxel allowed, avoiding the sinuses and ventricles. Due to the shapes of some brains, the shim volume centered on the voxel penetrated the ventricles in a few cases to a similar extent in adults and children. All voxels were placed to avoid cerebro‐spinal fluid (CSF) and were, if necessary, slightly angled. Due to individual variations in anatomy, slight differences in the signal contribution from neighboring areas could not entirely be avoided. However, the voxel covered mainly the region of interest and therefore reflected neurotransmitter concentrations of functionally relevant areas. Note that the choice of voxel size is commonly used at 3T field strength or higher [Barker, et al., 2010; Mekle, et al., 2009] and results in an optimal trade‐off between spatial resolution and signal‐to‐noise ratio (SNR). The localized MRS sequence SPECIAL was used to acquire 128 averages with a TR = 4,000 ms, a TE = 8.5ms, a bandwidth of 2,000 Hz, and 4,096 points [Mekle, et al., 2009; Mlynarik, et al., 2006]. Water suppression was performed using VAPOR (variable power radio frequency pulses with optimized relaxation delays) [Tkac and Gruetter, 2005]. SPECIAL enables reproducible GABA (and glutamate) measurements, provided that spectra are of sufficiently high quality in terms of signal to noise ratio and line width (LW) [Near, et al., 2013], as was the case in our study (Supporting Information Table S1 for line widths for each brain region/age group). Other, more established methods such as MEGA –PRESS do not enable separation of glutamate and glutamine, and thus only enable reporting of a composite measure, such as glx (glutamate + glutamine). Measures of GABA obtained using MEGA‐PRESS are also known to contain a significant unwanted contribution from macromolecules (known as GABA+) [Edden, et al., 2012]. SPECIAL, on the other hand, does enable simultaneous measurement of glutamate, glutamine, and GABA, and may be less sensitive to macromolecule contamination since it uses the resonances of all three of GABA's methylene groups and includes prior knowledge of their fine peak splittings/couplings to achieve independence from overlying macromolecular and metabolite resonances [Mekle, et al., 2009; Near, et al., 2013].

The current research focused on face processing‐related changes in the IOG, rather than the fusiform gyrus (FG), as the IOG is a necessary part of the core face network [Cohen Kadosh, et al., 2010; Pitcher, et al., 2007, 2009; Rotshtein, et al., 2007; Schiltz and Rossion, 2006], which reliably exhibits developmental differences [Cohen Kadosh, et al., 2011, 2013b]. Moreover, MRS research requires anatomically defined regions, but most research on developmental differences in functional FG response has shown substantial variation in location [Cohen Kadosh and Johnson, 2007].

#### Voxel‐based morphometry analysis

A VBM analysis [Ashburner and Friston, 2000] was conducted to investigate developmental changes in gray and white matter tissue volumes in specific regions‐of‐interest (ROIs) in all participants using the SPM8 VBM8 toolbox (http://dbm.neuro.uni-jena.de/vbm/). First, each participant's structural T1 image was normalized to the standard T1 MNI template. Then segmentation was performed using prior tissue probability maps, and all scans were segmented into cerebro‐spinal fluid and gray and white matter using Jacobian determinants for the nonlinear warping modulation. All images were smoothed with an 8 mm full‐width, half‐maximum Gaussian kernel. Using MarsBaR toolbox (http://marsbar.sourceforge.net/), we then extracted the mean gray matter adjusted volumes in 10 mm radius ROIs for the three MRS regions [right IPS (MNI (*x,y,z*)] = 50, −41, 52; left IFG = −46, 12, 18; right IOG = 33, −94, −6).

#### MRS analysis

Removal of motion corrupted spectral averages, and correction of frequency and phase drifts [Near, et al., in press] were performed in MATLAB (The Mathworks, Natick, MA) using the FID‐A toolkit (http://www.github.com/CIC-methods/FID-A) prior to signal averaging and data analysis. The Linear Combination (LC) Model [Provencher, 1993] with a simulated basis set including simulated macromolecule signal was used to analyze MRS data. The primary MRS outcome measure was the ratio of glutamate to GABA, and secondary outcome measures included the levels of the individual neurochemicals glutamate, GABA, glutamine, and *N*‐acetyl aspartate (NAA). In the case of the secondary outcome measures, all individual neurochemical concentrations were referenced to total creatine (creatine + phosphocreatine). Creatine referencing was used for the following three reasons: (1) The gray matter/white matter/cerebrospinal fluid (CSF) compartmentalization of creatine is similar to that of the metabolites of interest (high concentration in tissue vs low concentration in CSF). This is in contrast to water, which could serve as a reference, but unlike the neurotransmitters, has a high concentration in CSF. Therefore, creatine ratios are less dependent on the CSF volume fraction within the voxel, and do not necessarily require corrections to account for this (we note though that we have included these corrections as well). (2) Since the creatine signal is acquired simultaneously with the metabolites of interest, it is equally affected by experimental imperfections such as frequency drift, phase drift, and subject motion, and all processing steps affect these resonances equally. (3) Creatine is a commonly used and widely accepted internal reference standard, and we had no reason to believe that creatine levels would be related to any of the cognitive measures.

**Figure 2:**
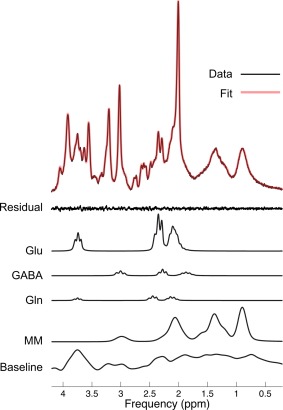
Decomposed example spectrum of an IFG voxel from the linear combination model in a child participant. The *x*‐axis shows the proton spin frequency expressed in parts per million (ppm) and the signal intensity is mapped along the *y*‐axis. The residual is the noise subtracted from the signal (Data), here demonstrating a good fit (low level of noise). Glutamate (Glu) and GABA were the metabolites of interest with three separate peaks each. Gln (glutamin) and MM (macromolecules) are shown here, as well as the simulated baseline to demonstrate the relationships between the measures. [Color figure can be viewed in the online issue, which is available at http://wileyonlinelibrary.com.]

Spectral quality was ascertained by small spectral line widths of all acquired spectra (all below 6Hz) and the signal‐to‐noise ratios (SNR) per region. Cramer‐Rao lower bounds (CRLBs) varied across the three regions (Supporting Information Table S1 for all quality control measures, including gray matter volume), with CRLBs being slightly higher for GABA measures in the IOG (i.e., around 20%), which was because of the close proximity to the cortical edge and the ventricles. See also Figure 2 for an example spectrum.

## RESULTS

### Age‐Dependent Differences in Task Performance and Brain Excitability and Structure

We found that children performed significantly worse than the adults on the Cambridge face memory test (CMFT), a computer‐based face recognition task [children = 63% correct (*z*‐score = −0.433); adults = 72% correct (*z*‐score = 0.452); group difference: *t*(25) = −2.47, *P* = 0.02, bootstrapped 95% confidence interval (CI) [−1.624, −0.146]]. The two groups also differed in their VS‐WMT scores; again, as expected, children achieved lower scores than the adults [children *z*‐score = −0.513; adults *z*‐score = 0.516; groups difference: *t*(25) = −3.02, *P* = 0.006, bootstrapped 95% CI [−0.176, −0.301].

We then assessed region‐specific glutamate/GABA ratios, and revealed a significant main effect of age on these ratios [*F*(1,44) = 13.50, *P* < 0.001, *ηp^2^* = 0.38], as well as an interaction between brain region and age [*F*(2,44) = 4.08, *P* = 0.045, *ηp^2^* = 0.16] (Fig. 1). Further analyses showed that this interaction was because of a significantly higher glutamate/GABA ratio in the IFG in the children than in the adults [*t*(25) = 3.87, *P* < 0.001, bootstrapped 95% CI [0.719, 2.36]]. The observed group difference in the IFG remained significant when we controlled for *a priori* group differences in line‐width [*F*(2,26) = 7.01, *P* = 0.004, *ηp^2^* = 0.379] or gray matter volume [*F*(3,26) = 6.61, *P* = 0.002, *ηp^2^* = 0.474]. No age group differences were found for the two other regions [IPS: *t*(25) = 0.737, *P* = 0.47, bootstrapped 95% CI [−0.937, 1.983]; IOG: *t*(22) = −1.61, *P* = 0.12, bootstrapped 95% CI [−8.57, 1.08]]. We further assessed group differences in gray matter volumes in the three MRS voxels and found that whereas gray matter volumes in the IFG voxel did not differ between children and adults [*t*(25) = −0.024, *P* = 0.981, bootstrapped 95% CI [−0.055, 0.053]], children had significantly more gray matter in the IPS voxel [*t*(25) = 6.24, *P* < 0.001, bootstrapped 95% CI [0.071, 0.141]] and the IOG voxel [*t*(25) = 2.84, *P*=0.009, bootstrapped 95% CI [0.019, 0.120]].

### Neurotransmitter Ratios Predict Face Processing Performance in the Children

To allow for a rigorous statistical assessment we computed 95% CIs for correlation coefficients based on 10,000 samples with the nonparametric, bias‐corrected, and accelerated percentile bootstrap method. All the correlation coefficients that we report as significant have survived the Benjamini‐Hochberg correction for multiple correlations [Lesack and Naugler, 2011]. Prior to the correlation analysis, we used an adjusted boxplot rule [Pernet, et al., 2012] to detect bivariate outliers. Given the robustness of Spearman's rho (used because of non‐normally distributed data) to outliers and the fact that the results did not differ between the two analysis approaches, correlation analyses are shown for the sample where the outliers are included. The results also remained the same when we controlled for group‐differences in MRS signal quality indices [line‐width (LW) or removed outliers based on the signal to noise ratio (SNR) or Cramer‐Rao lower bounds (CRLBs)].

**Figure 3:**
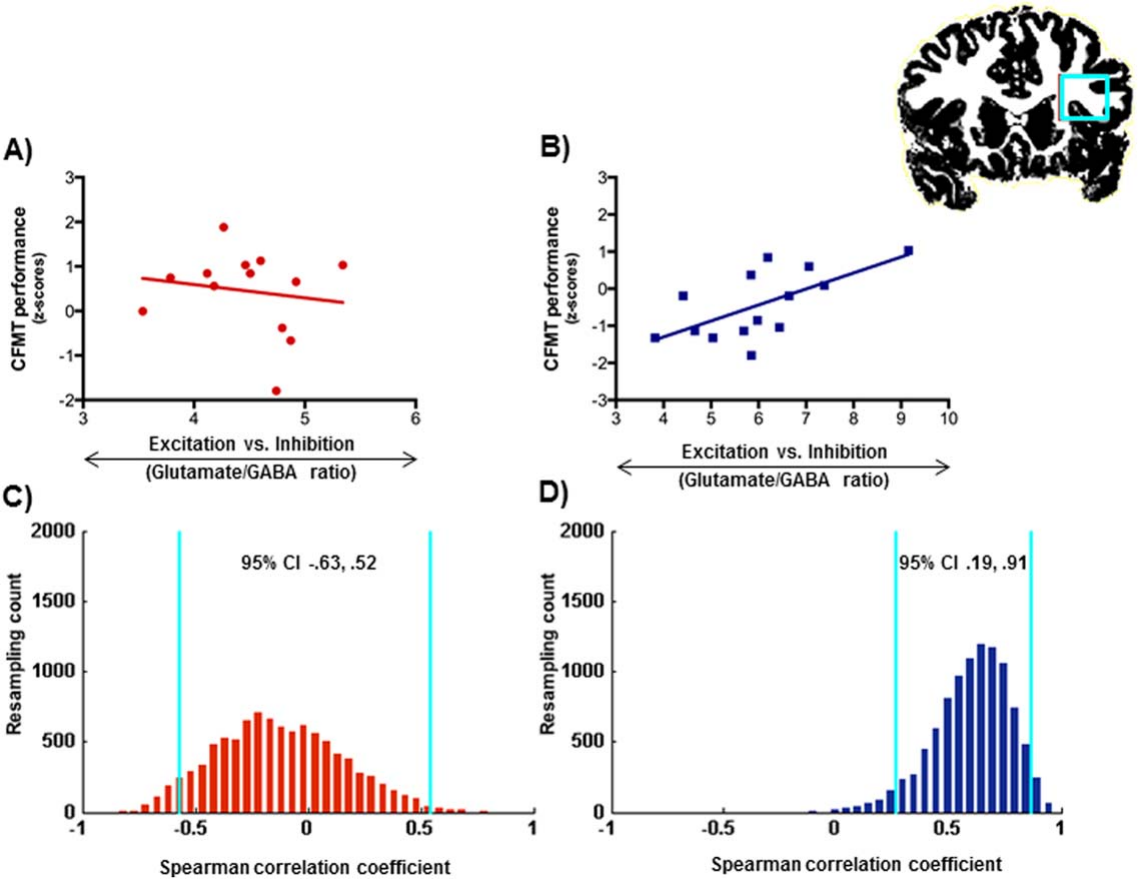
A, B: Relationship between cortical glutamate/GABA ratios and performance in the Cambridge face memory test (CFMT) in the inferior frontal gyrus in the adults (A) and the children (B). **C, D**: Bootstrap resampling data (blue lines indicate 95% CIs) for the correlations between glutamate/GABA ratios and performance in the CFTM in the adults (C) and the children (D). [Color figure can be viewed in the online issue, which is available at http://wileyonlinelibrary.com.]

For the face processing task, we found a significant correlation between performance and cortical glutamate/GABA ratios in the IFG in the children [*r*_s_(14) = 0.650, *P* = 0.012, bootstrapped 95% CI [0.187, 0.908]], which remained significant when we controlled for individual gray matter volume: [*r*_s_(11) = 0.636, *P* = 0.019, bootstrapped 95% CI [0.078, 0.877]] (Fig. 3; Table 1 for full details on all correlations). No significant correlations were found for the other two brain regions in the children: IPS [*r*_s_(14) = −0.203, *P* = 0.487, bootstrapped 95% CI [−0.792, 0.521]] or the IOG [*r*_s_(14) = 0.187, *P* = 0.521, bootstrapped 95% CI [−0.489, 0.705]]. In the adults, face task performance did not correlate with glutamate/GABA rations in any of the brain regions studied (all ps>0.17, see Table 1). Furthermore, as predicted, the correlation coefficient in the IFG was significantly larger for children than adults [*P* = 0.03].

**Table 1:** Bootstrapped Spearman correlations for neurotransmitter glutamate/GABA ratios in the inferior frontal gyrus (IFG), intraparietal gyrus (IPS), inferior occipital gyrus (IOG), and the face processing task in the children (n = 14) and the adults (n = 13, except for the IOG where n = 10)

	IPS	IOG	CFMT
IFG	*r*_s_= 0.011, *P* = 0.970	*r*_s_= 0.002, *P* = 0.994	***r*_s_= 0.650, *P* = 0.012**
	CI 95%(−0.586, 0.604)	CI 95%(−0.502, 0.528)	**CI 95%(0.187, 0.908)**
IPS	1	*r*_s_= −0.068, *P* = 0.817	*r*_s_= −0.203, *P* = 0.487
		CI 95%(−0.570, 0.516)	CI 95%(−0.792, 0.476)
IOG			*r*_s_= 0.187, *P* = 0.521
			CI 95%(−0.489, 0.705)
IFG	*r*_s_ = 0.110, *P* = 0.721	*r*_s_= 0.006, *P* = 0.987	*r*_s_ *=* −0.113, *P* = 0.713
	CI 95%(−0.517, 0.653)	CI 95%(−0.753, 0.782)	CI 95%(−0.627, 0.524)
IPS		*r*_s_ = 0.479, *P* = 0.162	*r*_s_ = −0.121, *P* = 0.693
		CI 95%(−0.355, 1.00)	CI 95%(−0.692, 0.487)
IOG			*r*_s_= −0.468, *P* = 0.172
			CI 95%(−0.941, 0.235)

All ps are two‐tailed and significant effects at the 0.05 level.

Abbreviations: CFMT = Cambridge Face Memory Test.

### Neurotransmitter Ratios Correlate Negatively With Visuo‐Spatial Working Memory Performance in the Adults

**Figure 4:**
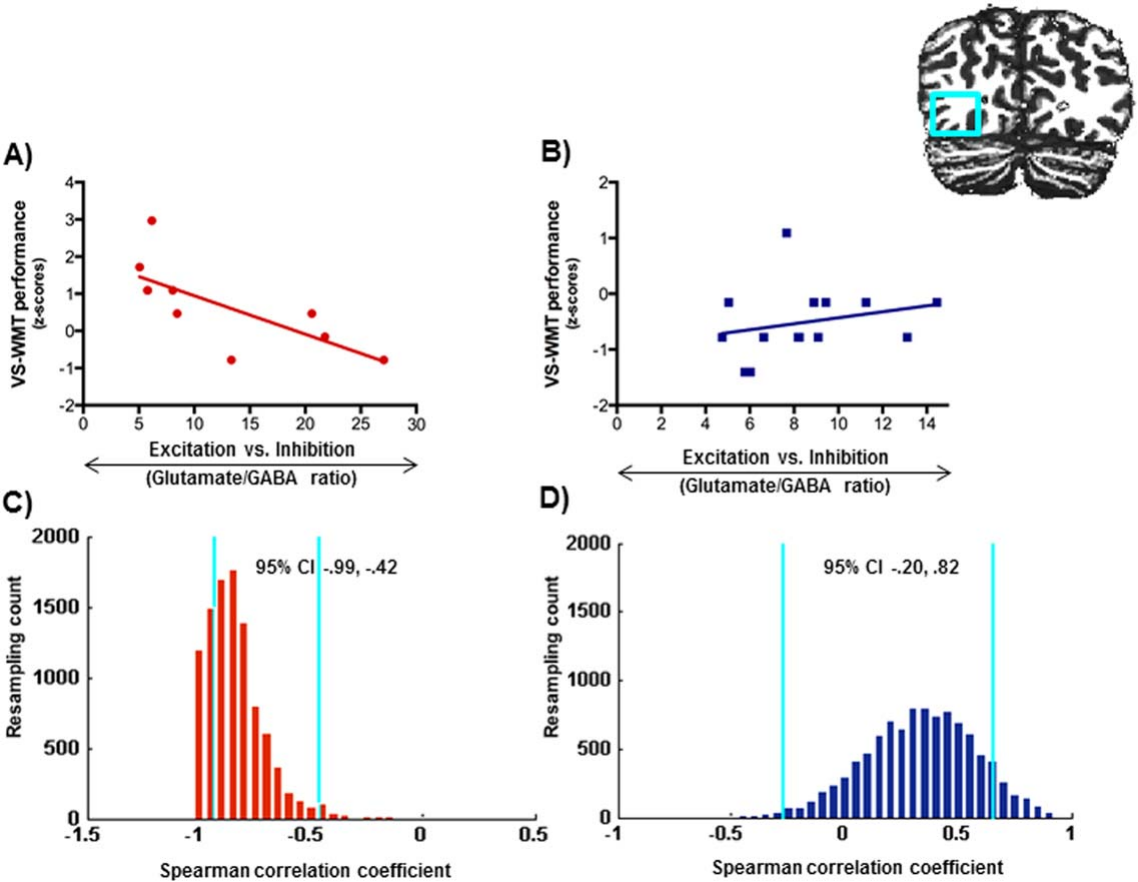
A, B: Relationship between cortical glutamate/GABA ratios and performance in the visuo‐spatial working memory task (VS‐WMT) in the inferior occipital gyrus in the adults (A) and the children (B). **C, D**: Bootstrap resampling data (blue lines indicate 95% CIs) for the correlations between glutamate/GABA ratios and performance in the VS‐WMT in the adults (C) and the children (D**)**. [Color figure can be viewed in the online issue, which is available at http://wileyonlinelibrary.com.]

We further examined whether the link between cortical glutamate/GABA ratios and cognition was domain‐ and age‐specific by correlating glutamate/GABA ratios with performance in the visuospatial working memory task (VS‐WMT) task. In the children we found no significant correlations for cortical glutamate/GABA ratios and the VS‐WMT task in any of the scanned brain regions (all ps > 0.20, see Table 2 for all correlations). In contrast, VS‐WMT performance in the adults correlated negatively with cortical glutamate/GABA ratios in the IOG [*r*_s_(10) = −0.84, *P* = 0.002, bootstrapped 95% CI [−0.994, −0.420]]. This effect was consistent and remained significant when we controlled for individual gray matter volume: [*r*_s_(7)= −0.764, *P* = 0.017, bootstrapped 95% CI [−0.996, −0.335], Fig. 4; Table 2]. No significant correlations were found for the IFG [*r*_s_(10) = −0.29, *P* = 0.43, bootstrapped 95% CI [−0.882, 0.541]] or the IPS [*r*_s_(10) = −0.29, *P* = 0.43, bootstrapped 95% CI [−0.882, 0.541]].

**Table 2:** Bootstrapped Spearman correlations for neurotransmitter glutamate/GABA ratios in the inferior frontal gyrus (IFG), intraparietal sulcus (IPS), inferior occipital gyrus (IOG), and the visuo‐spatial working memory task (VS‐WMT) in the children (n = 14) and in the adults (n = 13, except for the IOG where n = 10)

	IPS	IOG	VS‐WMT
IFG	*r*_s_= 0.011, *P* = 0.970	*r*_s_ = 0.002, *P* = 0.994	*r*_s_= 0.038, *P* = 0.899
	CI 95%(−0.586, 0.604)	CI 95%(−0.502, 0.528)	CI 95%(−0.670, 0.667)
IPS	1	*r*_s_ *=* −0.068, *P* = 0.817	*r*_s_= −0.153, *P* = 0.603
		CI 95%(−0.570, 0.516)	CI 95%(−0.615, 0.399)
IOG			*r*_s_= 0.361, *P* = 0.204
			CI 95%(−0.198, 0.820)
IFG	*r*_s_ = 0.110, *P* = 0.721	*r*_s_= 0.006, *P*= 0.987	*r*_s_= −0.022, *P* = 0.942
	CI 95%(−0.517, 0.653)	CI 95% (−0.753, 0.782)	CI 95% (−0.788, 0.685)
IPS		*r*_s_ = 0.479, *P* = 0.162	*r*_s_= −0.414, *P* = 0.160
		CI 95% (−0.355, 1.00)	CI 95% (−0.818, 0.202)
IOG			***r*_s_= −0.840, *P* = 0.002**
			**CI 95%(−0.994, −0.420)**

All ps are two‐tailed and significant effects at the 0.05 level.

### Effects of Single Neurotransmitter Concentrations

Finally, to gain some further insights into whether glutamate or GABA was the main driving source of the observed effects, we conducted additional analyses looking at each neurotransmitter concentration separately in relation to total Creatine levels. For the glutamate analysis we found that changes in glutamate concentrations alone in the IFG did not correlate significantly with performance in the CWMT [*r*_s_(14) = 0.518, *P* = 0.058, bootstrapped 95% CI [−0.053, 0.855]], and neither did GABA concentrations alone [*r*_s_(14) = −0.322, *P* = 0.262, bootstrapped 95% CI [−0.827, 0.273]]. This result suggests that the effect was dependent on glutamate/GABA ratios rather than glutamate only concentrations. In contrast, only for adults the IOG GABA was correlated with the VS‐WMT [*r*_s_(10) = 0.691, *P* = 0.009, bootstrapped 95% CI [0.229, 0.928]]. This positive correlation is in line with the findings of glutamate/GABA ratio; showing that in the case of the adults’ IOG, increase in GABA concentrations, rather than glutamate/GABA is the driving source of the observed decrease in VS‐WMT performance (Supporting Information Tables S2, S3 for all effects).

## DISCUSSION

In this study, we highlight the important contributions that a neurochemical research approach can bring to understanding typical development. Specifically, we investigated how changes in cognitive performance relate to functional and anatomical brain development. We found that higher levels of glutamate/GABA ratios in the IFG correlated with face processing proficiency in the children, but not the adults. That is, the higher the excitability levels in the IFG, the better the children performed in a face recognition task. This suggests that increased glutamate/GABA ratios in this brain region are associated with successful face processing at a relatively early developmental stage. Moreover, the fact that we show that is this effect was dissociated from underlying structural development. Considering that the frontal cortices in our young adult group are still exhibiting developmental change [Gogtay, et al., 2004; Tamnes, et al., 2013], this suggests that differences in neurotransmitter ratios may precede learning and structural change during development. This interpretation is further supported by the fact that the other, earlier maturing brain regions (IOG, IPS) in the young adults showed clear differences in cortical gray matter volume but without differences in excitability ratios.

We found a positive correlation between glutamate/GABA ratios and face processing proficiency in children only for the IFG, but not for the IOG, a core face network region that has often been reported in both children and adults [Cohen Kadosh, et al., 2011; Fairhall and Ishai, 2007; Rotshtein, et al., 2007]. Together, these results lead to the hypothesis that while high ratios of glutamate/GABA in the IFG may support the acquisition of face processing abilities, they are not necessary for proficient processing in the mature core network regions. The results also seem to indicate that the observed developmental changes were domain‐specific for face processing abilities. That is, when we examined VS‐WMT, which has been shown to activate other brain regions, and involves different cognitive architectures and developmental trajectories [Dumontheil and Klingberg, 2012; Klingberg, 2006; Kwon, et al., 2002], we found that the IOG glutamate/GABA ratio correlated negatively with VS‐WMT performance in the adult group, but not in the children. This finding seems to suggest that the maturation of glutamate/GABA ratio can create a critical “bottleneck” during development that constrains behavioral performance in a task. This interpretation was further supported in our additional analyses, where we found that whereas specific glutamate/GABA ratios in the IFG were related to good face processing performance during development, in the adults, the correlation between neurotransmitter levels and VS‐WMT performance remained significant when we looked at GABA concentration only. One tentative interpretation of these results might be that high levels of inhibition support VS‐WMT performance in the mature brain (i.e. the results of the GABA concentration levels only). However, it may be a specific balance in neurotransmitters that enables the acquisition of a new skill during development.

The finding that a particular brain region is associated with the acquisition of a cognitive skill at a specific developmental stage runs in line with neuroconstructivist theories of brain development, such as the Interactive Specialization framework [Johnson, 2001]. These approaches propose that new behavioral competencies emerge as a result of slowly specializing cortical networks, a process that is guided by “architectural constraints” [Elman, et al., 1996]. These architectural constraints are expressed as slight differences in the patterns of intrinsic connectivity, synaptic density, and, most important for the current study, the balance of neurotransmitters and the way they interact [Johnson, 2011; Johnson, et al., 2002]. It has further been suggested that these constraints are reflected in the changing response functions of different brain regions during the acquisition of a new cognitive ability, such as face processing [Johnson, et al., 2009]. Our work corroborates previous neuroimaging findings at the functional level that documented changing functional responses in the IFG during face processing tasks. That is, we demonstrate that it is feasible to link cognitive processing abilities to individual differences at the neurochemical level and more specifically in glutamate/GABA ratios in the developing human brain, using noninvasive brain imaging techniques. This neurochemical approach also allowed us to shed new light on developmental differences in the IFG when more traditional structural assessment techniques might not yet detect group differences between children and young adults because of the protracted maturational trajectory of the frontal lobe [Gogtay, et al., 2004; Tamnes, et al., 2013].

Animal studies have shown that the response properties of cortical neurons are determined by interactions between their excitatory and inhibitory synaptic inputs [Oswald, et al., 2006; Zhang, et al., 2011]. Although many of those properties originate subcortically, interactions between synaptic excitation and inhibition in the cortex can refine the stimulus tuning of the neurons found there or even create new feature selectivity. The balance between excitation and inhibition is therefore critical for normal cortical function. In the context of the present study, there is evidence that the balance of excitation and inhibition changes, however, during the course of postnatal development, reflecting refinements either in excitatory input strengths [Sun, et al., 2010] or in intracortical inhibition [Dorrn, et al., 2010]. The initial unbalanced excitation is thought to allow activity‐dependent refinements of cortical circuits [Carcea and Froemke, 2013]. Early sensory experience has been shown to improve the coupling between excitatory and inhibitory inputs, which appears to limit subsequent plasticity in the cortex [Dorrn, et al., 2010]. In a similar vein, we found here that glutamate/GABA ratios changed with age in the IFG, and that these changes were correlated with the involvement of this area in face processing in children but not in adults. Developmental regulation of glutamate/GABA ratios may therefore determine the timing of sensitive periods for the acquisition of perceptual and cognitive skills [Dorrn, et al., 2010; Hensch and Bilimoria, 2012; Toyoizumi, et al., 2013], while deficits in this process are associated with neurodevelopmental disorders, such as autism [Gogolla, et al., 2009] and schizophrenia [Insel, 2010].

As with any other noninvasive neuroimaging methods in human participants, 1H‐MRS offers a relatively coarse spatial resolution (centimeter) compared with studies that examine glutamate/GABA ratios at the level of neurons and neural circuits in animals. Furthermore, MRS glutamate measures may not necessarily be specific to the pool of excitatory glutamate. That is, as with previous MRS research, it is unclear to what extent our measurement quantifies metabolic vs. neurotransmitter glutamate pools. However, a comparative analysis of *N*‐acetylaspartate (NAA), a marker of neuronal viability in the same brain regions did not show the same results (e.g., NAA/Creatine (Cr) ratios vs. face processing performance in the children in the IFG: [*r*_s_(14) = −0.212, *P* = 0.468, bootstrapped 95% CI [−0.868, 0.483]]; or NAA/Cr ratios vs. VS‐WMT performance in the adults in the IOG: [*r*_s_(11) = −0.042, *P* = 0.903, bootstrapped 95% CI [−0.725, 0.686]]). In addition, we also looked at resting levels of glutamine (both against total levels of Creatine or as glutamine/GABA ratios), based on the previously made suggestion that “total glutamate levels could be a reasonable metric of glutamate involved in glutamatergic neurotransmission” [Hu, et al., 2013]. The results for both analyses showed that glutamine levels did not correlate with face processing performance in the children in the IFG or with VS‐WMT performance in the adults (Supporting Information Tables S4, S5 for all analyses). Based on that, we suggest that the observed correlations were likely driven more by the metabolic pool, rather than the neurotransmitter pool of glutamate. However, we would like to point out that MRS measures of glutamine are less reproducible than glutamate measures due to the relatively small levels of glutamine in the brain. Future studies may shed further light on the contribution of metabolic vs. neurotransmitter pools to cognitive, skills acquisition, and development in humans.

Either way, the observed relationships between neurotransmitter ratios and cognitive abilities in our study are corroborated by animal findings at the cellular level [Majdi, et al., 2009; Yizhar, et al., 2011] and provide the necessary extension to humans. Future research is now needed to optimize ^1^H‐MRS parameters for use with human adult and paediatric populations and to increase spatial and temporal resolution for simultaneous measurements of glutamate and GABA. Such advancements will also help shed light on how cortical glutamate/GABA ratios in humans relate to the finer measurements that can be made in animal models, an approach which has already been used in the development and advancement of other noninvasive neuroimaging techniques, such as functional magnetic resonance imaging [Logothetis, 2007]. We also note that the current study was done in females only to control for any gender‐related variation in the MRS signal, an approach that is common in the field of developmental cognitive neuroscience. However, even such a careful approach does not preclude the possibility of variations in neurotransmitter ratios across the menstrual cycle in the older women [Smith, et al., 2002] see also [Krause and Cohen Kadosh, 2014].

## CONCLUSIONS

The current study investigated the changing relationship between excitatory and inhibitory neurotransmitter concentrations in predefined cortical regions and cognitive abilities, namely face processing proficiency and VS‐WMT in children and adults. Our results suggest that while increased glutamate/GABA ratios may be crucial for acquiring new cognitive skills and neuroplasticity during development, maintaining these levels of neurochemical ratios may not be necessary for proficient functioning in later life. Namely, in our case cognitive performance in adults was better characterized by GABA, rather than the ratio between glutamate and GABA. A better understanding of how these emerging brain networks for cognitive functions become ‘hardwired’ can then guide the search for new brain‐based biomarkers that indicate cognitive and brain plasticity in a given period. The current work also highlights the value of investigating the relationship between neurotransmitters, such as GABA and glutamate, brain anatomy and processing, and cognition across the lifespan.

## Supporting information

Supporting Information
