# Supplementary material for: Linking GABA and glutamate levels to cognitive skill acquisition during development
Source: Hum Brain Mapp. 2015 Sep 9;36(11):4334–45. doi: 10.1002/hbm.22921 (PMC4832309; doi:10.1002/hbm.22921)
Supplement: Supplementary file 1 — Supporting Information [file HBM-36-4334-s001.docx]

**Table S1.** *Means, range and standard deviations of gray matter volume, the Cramer-Rao lower bounds, and line widths for the inferior frontal gyrus (IFG), intraparietal gyrus (IPS), inferior occipital gyrus (IOG) in the children (n= 14; except for line width where: n=12) and the adults (n= 13, except line width where: n=12). Bold t-tests and bootstrapped confidence intervals indicate mean group difference.*

| ***Gray matter (mean, range and standard deviations)*** | | | | |  |  |  |
| --- | --- | --- | --- | --- | --- | --- | --- |
|  | **Children** | **Adults** | **t-test** | |  |  |  |
| **IFG** | .555 (.39-.71)  .082 | .555 (.44 - .63)  .049 | *t*(25)= -.024, *p*= .981, *CI95%*(-.055, .053) | |  |  |  |
| **IPS** | .567 (.52-.69)  .045 | .461 (.40 - .55)  .043 | ***t*(25)= 6.24, *p*< .001, *CI95%*(.071, .141)** | |  |  |  |
| **IOG** | .366 (.25-.49)  .078 | .297 (.24 - .37)  .017 | ***t*(25)= 2.84, *p*= .009, *CI95%*(.019, .120)** | |  |  |  |
|  | **GABA** |  |  | | **Glutamate** | |  |
| ***Cramer-Rao lower bounds (mean, range and standard deviations)*** | | | | |  | |  |
|  | **Children** | **Adults** | **t-test** | | **Children** | **Adults** | **t-test** |
| **IFG** | 15.5 (13-20)  2.11 | 13.4 (11-17)  1.83 | ***t*(23)= 2.58, *p*= .017, *CI95%*(.405, .368)** | | 4.0 (3-5)  0.41 | 4.0 (3-5)  0.43 | *t*(23)= 0.00, *p*= 1.0, *CI95%*(-.345, .345) |
| **IPS** | 16.5 (12-24)  3.85 | 17.3 (14- 25)  3.35 | *t*(25)= -.998, *p*= .328, *CI95%*(-4.23, 1.46) | | 4.0 (4-4)  0.00 | 3.9 (3-4)  0.28 | *t*(25)= .052, *p*= .959, *CI95%*(-.210, .221) |
| **IOG** | 21.6 (16-35)  6.19 | 21.8 (17-29)  5.50 | *t*(13)= -.030, *p*= .976, *CI95%*(-8.19, 7.96) | | 4.4 (4-5)  0.51 | 4.3 (4-5)  0.51 | *t*(13)= .677, *p*= .510, *CI95%*(-.448, .857) |
| ***Line width (mean, range and standard deviations)*** | | | | |  |  |  |
| **IFG** | 3.6 (2.95-4.92)  .608 | 4.09 (3.45-4.92)  .544 | | *t*(23)= 0.00, *p*= 1.0, *CI95%*(-.345, .345) |  |  |  |
| **IPS** | 2.99 (2.46-3.94)  .492 | 3.10 (2.95-3.45)  .240 | | *t*(25)= .773, *p*= .447, *CI95%*(-.428, .194) |  |  |  |
| **IOG** | 3.70 (2.95-5.41)  .610 | 4.34 (3.45-5.91)  .689 | | ***t*(13)= 3.15, *p*= .008,**  ***CI95%*(-.1.09, -2.03)** |  |  |  |

***Table S2.*** *Bootstrapped* *Spearman correlations for neurotransmitter glutamate concentrations (referenced to total Creatine) in the inferior frontal gyrus (IFG), intraparietal gyrus (IPS), inferior occipital gyrus (IOG) and the face processing task (CFMT) and the Visuo-spatial working memory task (VS-WMT) in the children (n= 14) and the adults (n= 13, except for the IOG where n=10). All ps are two-tailed and significant effects at the .05 level (uncorrected for multiple comparisons). Abbreviations: CFMT= Cambridge Face Memory Test.*

|  | **IPS** | | **IOG** | **CFMT** | **VS-WMT** |
| --- | --- | --- | --- | --- | --- |
| **IFG** | | *r_s_*= .380, *p*= .180 | *r_s_=* .051, *p*= .864 | *r_s_=* .518, *p*= .058 | *r_s_=* .068, *p*= .817 |
|  | | *CI95%*(-.011, .304) | *CI95%*(-.571, .672) | CI95%(-.053, .855) | CI95%(-.553, .624) |
| **IPS** | |  | *r_s_=* .068, *p*= .817 | *r_s_=* .276, *p*= .340 | *r_s_=* -.134, *p*= .648 |
|  | |  | *CI95%*(.000, .300) | *CI95%*(-.507, .795) | *CI95%*(-.712, .461) |
| **IOG** | |  |  | *r_s_=* .176, *p*= .546 | *r_s_=* .265, *p*= .359 |
|  | |  |  | *CI95%*(-.445, .593) | *CI95%*(-.366, .697) |
| **IFG** | | *r_s_*= -.088, *p*= .755 | *r_s_*= -.063, *p*= .837 | *r_s_=* -.179, *p*=.558 | *r_s_=* -.353, *p*=.237 |
|  | | *CI95%*(-.649, .597) | *CI95%*(-.657, .641) | *CI95%*(-.697, .499) | *CI95%*(-.763, .263) |
| **IPS** | |  | *r_s_*= .545, *p*= .054 | *r_s_=* .419, *p*=.154 | *r_s_=* .000, *p*= 1.00 |
|  | |  | *CI95%*(-.118, .881) | *CI95%*(-.226, .807) | *CI95%*(-.532, .546) |
| **IOG** | |  |  | *r_s_=* -.153, *p*=.618 | *r_s_=* -.200, *p*=.512 |
|  | |  |  | *CI95%*(-.417, .632) | *CI95%*(-.718, .474) |

***Table S3.*** *Bootstrapped* *Spearman correlations for neurotransmitter GABA concentrations (vs. total Creatine) in the inferior frontal gyrus (IFG), intraparietal gyrus (IPS), inferior occipital gyrus (IOG) and the face processing task (CFMT) and the Visuo-spatial working memory task (VS-WMT) in the children (n= 14) and the adults (n= 13, except for the IOG where n=10). All ps are two-tailed and significant effects at the 05 level (uncorrected for multiple comparisons). Abbreviations: CFMT= Cambridge Face Memory Test.*

|  | **IPS** | | **IOG** | **CFMT** | **VS-WMT** |
| --- | --- | --- | --- | --- | --- |
| **IFG** | | *r_s_*= .235, *p*= .418 | *r_s_=* -.253, *p*= .383 | *r_s_=* -.322, *p*= .262 | *r_s_=* -.019, *p*= .949 |
|  | | *CI95%*(-.479, .731) | *CI95%*(-.739, .388) | CI95%(-.827, .273) | CI95%(-.571, .576) |
| **IPS** | | 1 | *r_s_=* -.095, *p*= .748 | *r_s_=* .362, *p*= .204 | *r_s_= .*061, *p*= .836 |
|  | |  | *CI95%*(-.609, .554) | *CI95%*(-.251, .840) | *CI95%*(-.492, .566) |
| **IOG** | |  |  | *r_s_= -*.176, *p*= .546 | *r_s_=* .-427, *p*= .128 |
|  | |  |  | *CI95%*(-.798, .591) | *CI95%*(-.893, .171) |
| **IFG** | | *r_s_*= .022, *p*= .943 | *r_s_*= -.226, *p*= .459 | *r_s_=* -.047, *p*=.879 | *r_s_=* -.316, *p*=.293 |
|  | | *CI95%*(-.634, .692) | *CI95%*(-.812, .433) | *CI95%*(-.640, .536) | *CI95%*(-.811, .304) |
| **IPS** | |  | *r_s_*= .490, *p*= .089 | *r_s_=* .328, *p*=.274 | *r_s_=* .420, *p*= .153 |
|  | |  | *CI95%*(-.158, .874) | *CI95%*(-.309, .837) | *CI95%*(-.188, .775) |
| **IOG** | |  |  | *r_s_=* .366, *p*=.219 | ***r_s_= .*691, *p*=.009** |
|  | |  |  | *CI95%*(-.221, .774) | ***CI95%*(.229, .928)** |

***Table S4.*** *Bootstrapped* *Spearman correlations for neurotransmitter glutamine concentrations (vs. total Creatine) in the inferior frontal gyrus (IFG), intraparietal gyrus (IPS), inferior occipital gyrus (IOG) and the face processing task (CFMT) and the Visuo-spatial working memory task (VS-WMT) in the children (n= 14) and the adults (n= 13, except for the IOG where n=10). All ps are two-tailed and significant effects at the 05 level (uncorrected for multiple comparisons). Abbreviations: CFMT= Cambridge Face Memory Test.*

|  | **IPS** | | **IOG** | **CFMT** | **VS-WMT** |
| --- | --- | --- | --- | --- | --- |
| **IFG** | | *r_s_*= .169, *p*= .563 | *r_s_=* -.187, *p*= .523 | *r_s_=* .322, *p*= .262 | *r_s_=* .009, *p*= .975 |
|  | | *CI95%*(-.519, .780) | *CI95%*(-.688, .374) | CI95%(-.298, .789) | CI95%(-.540, .566) |
| **IPS** | | 1 | *r_s_=* -.204, *p*= .483 | *r_s_=* .373, *p*= .189 | *r_s_=* -.397, *p*= .160 |
|  | |  | *CI95%*(-.691, .396) | *CI95%*(-.276, .854) | *CI95%*(-.797, .206) |
| **IOG** | |  |  | *r_s_=* -.357, *p*= .210 | *r_s_=* .540, *p*= .046 |
|  | |  |  | *CI95%*(-.838, .270) | *CI95%*(-.117, .916) |
| **IFG** | | *r_s_*= .209, *p*= .494 | *r_s_*= -.470, *p*= .105 | *r_s_=* .220, *p*=.469 | *r_s_=* .120, *p*=.695 |
|  | | *CI95%*(-.526, .802) | *CI95%*(-.866, .143) | *CI95%*(-.371, .769) | *CI95%*(-.457, .728) |
| **IPS** | |  | *r_s_*= .249, *p*= .413 | *r_s_=* .030, *p*=.922 | *r_s_=* .000, *p*= 1.00 |
|  | |  | *CI95%*(-.392, .806) | *CI95%*(-.552, .590) | *CI95%*(-.689, .693) |
| **IOG** | |  |  | *r_s_=* -.105, *p*=.732 | *r_s_=* -.203, *p*=.507 |
|  | |  |  | *CI95%*(-.500, .617) | *CI95%*(-.427, .669) |

***Table S5.*** *Bootstrapped* *Spearman correlations for neurotransmitter glutamine concentrations (vs. GABA) in the inferior frontal gyrus (IFG), intraparietal gyrus (IPS), inferior occipital gyrus (IOG) and the face processing task (CFMT) and the Visuo-spatial working memory task (VS-WMT) in the children (n= 14) and the adults (n= 13, except for the IOG where n=10). All ps are two-tailed and significant effects at the 05 level (uncorrected for multiple comparisons). Abbreviations: CFMT= Cambridge Face Memory Test.*

|  | **IPS** | | **IOG** | **CFMT** | **VS-WMT** |
| --- | --- | --- | --- | --- | --- |
| **IFG** | | *r_s_*= .253, *p*= .383 | *r_s_=* -.393, *p*= .164 | *r_s_=* .434 *p*= .121 | *r_s_=* .056, *p*= .848 |
|  | | *CI95%*(-.294, .721) | *CI95%*(-.843, .275) | CI95%(-.204, .879) | CI95%(-.508, .624) |
| **IPS** | | 1 | *r_s_=* .007, *p*= .982 | *r_s_=* -.053, *p*= .857 | *r_s_=* -.303, *p*= .293 |
|  | |  | *CI95%*(-.555, .551) | *CI95%*(-.645, .568) | *CI95%*(-.766, .238) |
| **IOG** | |  |  | *r_s_=* -.238, *p*= .412 | *r_s_=* .526, *p*= .054 |
|  | |  |  | *CI95%*(-.750, .317) | *CI95%*(-.100, .905) |
| **IFG** | | *r_s_*= .302, *p*= .316 | *r_s_*= -.221, *p*= .468 | *r_s_=* .127, *p*=.680 | *r_s_=* .341, *p*=.254 |
|  | | *CI95%*(-.362, .731) | *CI95%*(-.762, .407) | *CI95%*(-.459, .639) | *CI95%*(-.364, .808) |
| **IPS** | |  | *r_s_*= .033, *p*= .914 | *r_s_=* -.163, *p*= .596 | *r_s_=* -..255, *p*= .401 |
|  | |  | *CI95%*(-.680, .635) | *CI95%*(-.826, .563) | *CI95%*(-.730, .432) |
| **IOG** | |  |  | *r_s_=* -.352, *p*=.238 | *r_s_=* -.422, *p*=.151 |
|  | |  |  | *CI95%*(-.772, .260) | *CI95%*(-.797, .346) |
